# Integrity as a Control Problem: Smooth and Adaptive Protection Levels for Multi-Modal Localization

**DOI:** 10.3390/s26165140

**Published:** 2026-08-14

**Authors:** Elias Maharmeh, Paulo Resende, Fawzi Nashashibi

**Affiliations:** 1 VALEO DAR—Driving Assistance Research, Valeo Mobility Tech Center (VMTC), 6 Rue Daniel Costantini, 94000 Créteil, France; paulo.resende@valeo.com; 2 Inria—ASTRA Team, 48 Rue Barrault, 75013 Paris, France; fawzi.nashashibi@inria.fr

**Keywords:** protection level, integrity monitoring, PID control, sensor fusion, urban navigation, integrity risk

## Abstract

Protection levels for autonomous vehicle localization are traditionally derived from estimator covariances under Gaussian assumptions. These approaches fail in complex urban environments where sensor anomalies produce heavy-tailed, non-Gaussian error distributions. This paper presents a fundamentally different paradigm that reformulates integrity monitoring as a closed-loop control problem. The method computes an instantaneous error rate from three sources: inertial sensor noise, kinematic drift between filter-based and dead-reckoned displacement, and LiDAR scan-map registration quality weighted by a sensitivity factor. This rate drives a saturation-controlled setpoint dynamics, then an adaptive PID controller with entropy-based gain scheduling produces the final protection level. Asymmetric update laws enforce rapid expansion but cautious contraction of safety bounds. Experiments on three UrbanNavDataset sequences (medium-urban, low-urban, deep-urban) demonstrate that traditional covariance-based methods exhibit high integrity risk, while the proposed framework achieves 0.0% risk in moderate environments and 2.3% under extreme degradation. The resulting protection levels are smooth and well-behaved, compatible with modern motion planners. This control-theoretic approach offers a viable alternative to statistical integrity paradigms in challenging real-world conditions.

## 1. Introduction

Localization is a core perception task for autonomous vehicles and mobile robotic platforms [1,2,3,4,5]. Knowing the platform’s position in absolute global coordinates is a prerequisite for navigation, path planning, and motion control. Traditionally, localization systems have been evaluated solely based on accuracy: the closer the estimate to ground truth, the better the performance. However, accuracy alone is insufficient. From an integrity monitoring perspective, a localization system must also quantify its own uncertainty or error and provide a numerical bound on its potential error; a value known as the protection level (PL). The ability to produce such a bound is critical for downstream tasks including path planning and low-level control. Consequently, offering a reliable PL has become an essential feature of modern localization systems.

Conventional integrity monitoring for Global Navigation Satellite System (GNSS)-only localization follows a two-step process [6,7]: first, fault detection and exclusion; second, PL computation. In this work, we extend integrity monitoring to multi-modal sensor fusion systems that integrate LiDAR, inertial measurement unit, and GNSS. Under the true-error probability distribution $P_{\mathrm{true}\text{-}\mathrm{error}}\left(\cdot \right)$ of the position error (PE), the PL is defined as:

(1) $$P_{\mathrm{true}\text{-}\mathrm{error}}\left(\mathrm{PE}>\mathrm{PL}\right)<\mathrm{TIR}$$

where PE denotes the error between the localization system’s estimated state and the ground truth state, and TIR is the target integrity risk; which is defined as the acceptable probability that the event PE > PL occurs [8], typically specified per estimated state. The right-hand side of Equation (1) is termed the empirical Integrity Risk or Integrity Risk (IR). *The objective is to provide a PL that overbounds the position error with probability at least 1−TIR. We acknowledge that availability and continuity are standard metrics in navigation systems, where availability denotes the percentage of time the system is usable, while continuity reflects the probability of maintaining performance over a phase of operation [7,8]. However, these are not addressed in this work.*

In this work, we will follow a different deterministic approach rather than a probabilistic approach, where this work reframes integrity monitoring as a closed-loop control system instead of relying on covariance estimates that fail under non-Gaussian conditions. The approach computes an instantaneous error rate from three contributing factors: inertial sensor noise, the difference between filter-based and dead-reckoned displacement, and LiDAR scan-map registration quality weighted by a sensitivity factor derived from the Jacobian matrix. Rather than integrating this rate indefinitely (which would grow without bound), it is fed into a bounded differential equation controlled by a saturation function that depends on the current setpoint value. This produces a finite target bound called the setpoint.

The main innovation decouples this theoretical setpoint from the operational PL using a PID controller as a smoothing mechanism. When a GNSS signal causes the setpoint to drop abruptly, the PID controller generates smooth, continuous commands that prevent sudden jumps in the safety boundary. The controller adjusts itself based on environmental entropy computed from LiDAR residuals: in cluttered or feature-poor environments, the proportional gain increases for faster response while the integral and derivative gains decrease to avoid amplifying noise. Asymmetric dynamics are enforced so that the PL rises immediately when the setpoint increases but falls slowly when the setpoint decreases, requiring consistent evidence before the safety bound shrinks.

The result is a PL that remains smooth and well-behaved even under severe sensor disturbances. This transforms integrity monitoring from a passive statistical estimation task into an active control problem, producing safety bounds that absorb shocks, filter noise, and meet the smoothness requirements of modern motion planning systems where conventional covariance-based methods break down.

This paper makes three main claims. First, by modeling the instantaneous error rate, the proposed PID-based framework produces PLs that remain smooth and well-behaved even under severe sensor anomalies. Second, entropy-driven gain adaptation enables the controller to respond appropriately across diverse environments: aggressive in chaotic conditions, conservative in predictable ones. Third, asymmetric update dynamics ensure rapid response to increasing uncertainty while requiring sustained evidence before relaxing safety bounds.

## 2. Related Work

Integrity carries multiple definitions in the literature; we refer the reader to [9] for an in-depth discussion of the variations in integrity and PL definitions for multi-modal sensor localization systems. In this work, we adopt the definition that integrity represents *alignment with reality*; specifically, the ability of a localization system’s PL to conservatively reflect the actual errors experienced under a given scenario. Under this definition, a system exhibits high integrity when the estimated PL closely tracks the true error while maintaining a conservative overbound. We next review the primary methods for computing PL in multi-modal sensor localization systems. Notably, unlike the extensive body of work on GNSS integrity, research on PL computation for multi-modal localization remains limited.

Several approaches compute the PL as a function of the maximum eigenvalue of the state covariance matrix P, expressed as $\mathrm{PL}=K\cdot \sqrt{\max(\mathrm{eigenvalue}(P))}$. In [10,11], the Gaussian assumption is replaced with a Student-*t* distribution, and the constant *K* is derived from the Student-*t* inverse cumulative distribution function to account for heavy-tailed noise. Building on this, ref. [12] develops a full Student-*t* Kalman filter with adaptively selected *K* based on the prediction-update cycle. More recently, ref. [13] applies the same formulation under Gaussian assumptions but employs Bayesian optimization to compute the optimal measurement noise covariance. A common limitation across these methods is their reliance on a fault detection and exclusion step prior to fusion and PL computation to ensure the distributional assumptions hold at each time step.

Alternative strategies learn the true-error distribution directly. In [14,15], the parameters of the true-error distribution are optimized jointly with the state estimate, and the PL is derived using the cumulative density function [15] or conditional value-at-risk [14]. Similarly, ref. [16] models the true-error distribution as a Gaussian mixture parameterized by a convolutional neural network, then computes the PL from the mixture. However, learning-based methods face well-known challenges: they depend heavily on the training dataset, suffer from generalization issues, and when applied online, their performance is sensitive to the optimization window size. A distinct line of work [17,18] formulates the PL as a bounding box using zonotopes and stochastic reachability, yet these methods still require fault detection and exclusion as a prerequisite.

In contrast to all the approaches reviewed above, the method introduced in the next section takes a fundamentally different direction. Rather than relying on distributional assumptions (Gaussian, Student-*t*, or learned mixtures), fault detection steps, or covariance propagation, we re-frame the PL calculation as a closed-loop control problem. Our approach does not require the system to declare whether a measurement is faulty or nominal before computing the PL. Instead, it continuously tracks an instantaneous error rate derived from sensor measurements and LiDAR registration quality, feeds this rate into a bounded saturation dynamics to generate a target setpoint, and then uses an adaptive PID controller to produce a smooth, continuous PL.

However, a PID controller with static parameters is fundamentally insufficient for the objective of having a dynamic PL. Therefore, the controller parameters must adapt dynamically online. Established techniques for dynamic adaptation broadly fall into *Adaptive Control* and *Gain-Scheduled Control*. Adaptive control typically entails the online recursive estimation of unknown, unmodeled, or time-varying plant dynamics to continuously update the control law [19,20]. Conversely, Gain-Scheduled Control avoids online system identification by adjusting controller gains in real-time based on a measurable external scheduling variable that dictates the current operating regime [21,22,23]. Within this context, integrity naturally aligns with Gain-Scheduled Control. By utilizing environmental entropy as a continuous scheduling variable, the proposed controller dynamically schedules its PID gains.

In the next section, the localization system used in this paper is briefly introduced in order to have a coherent flow of information and for better understanding of the method.

## 3. Multimodal Sensor Fusion

**Figure 1:**
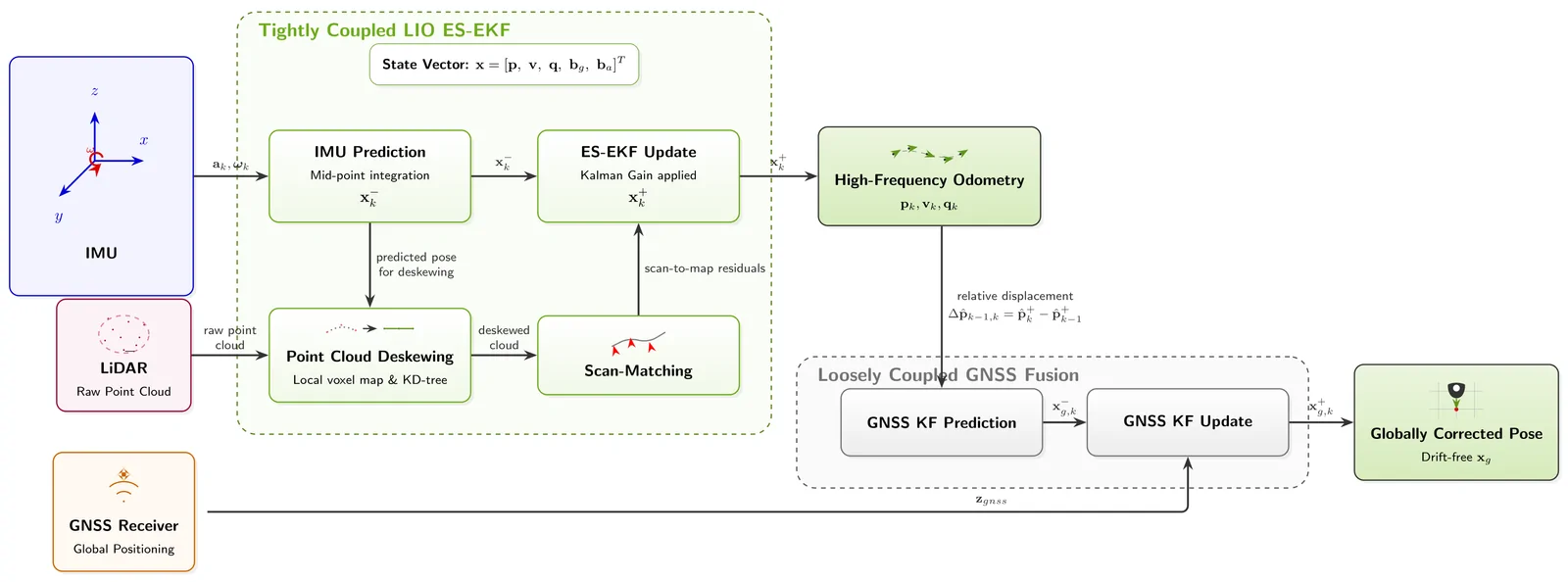
Architecture of the ES-EKF localization system.

This work builds upon the localization system introduced in [24], which performs a two-stage fusion process as illustrated in Figure 1. The first fusion level tightly couples IMU prediction with scan-map matching using Error-State Extended Kalman Filter (ES-EKF). The state vector is defined as $x=\left[p,v,\theta ,b_{g},b_{a}\right]\in R^{1\times 15}$, where $p\in R^{1\times 3}$ denotes position, $v\in R^{1\times 3}$ denotes velocity, $\theta \in R^{1\times 3}$ represents the minimal angle representation, and $b_{g},b_{a}\in R^{1\times 3}$ are the gyroscope and accelerometer biases in the body frame, respectively.

The state is propagated using IMU measurements through pure inertial navigation [25]. The predicted pose then deskews the incoming LiDAR scan and performs scan-map matching against the locally built map. The resulting residuals serve as tightly coupled innovations that update the local high-frequency state. To obtain global positioning—essential for autonomous vehicle navigation tasks—this high-frequency state is fused with GNSS measurements $z_{\mathrm{gnss}}$ using a standard Kalman filter. The final output comprises the global state estimate $x_{g}$ and its associated covariance matrix $P_{g}$. Readers seeking further details on the localization system are referred to [24,25,26,27,28].

## 4. The Proposed Approach

Most traditional PL frameworks rely predominantly on the internal covariance matrix of the state estimator to compute safety bounds. This paradigm implicitly assumes that the error distribution remains Gaussian across varying environmental conditions, that all sensor measurements are healthy, and that the reported covariances are well-calibrated. In practice, complex urban environments induce severe sensor anomalies that produce heavy-tailed, non-Gaussian error distributions. Within traditional frameworks, these anomalies manifest as abrupt, discontinuous jumps in the computed PL. Such discontinuities are problematic for autonomous motion planning algorithms [29], which benefit from smooth and continuous safety bounds to generate stable vehicle trajectories [30]. When the PL changes abruptly, the planner may respond with unnecessary braking or erratic steering corrections.

The proposed method resolves this by reformulating uncertainty bounding as a dynamic control problem. We decouple the mathematically ideal uncertainty bound from the operationally deployed safety bound, using a Proportional-Integral-Derivative (PID) framework to bridge the two. The following subsections detail the complete pipeline: projecting errors into the vehicle frame, quantifying physical sensor discrepancies, generating a saturated target bound, and robustly tracking it via adaptive control.

### 4.1. Lateral and Longitudinal Decomposition

We decompose the PLs into longitudinal (along-track) and lateral (cross-track) directions. Given the current heading estimate ${\hat{\psi }}_{k}$, the longitudinal and lateral basis vectors are defined as:

(2) $$\hat{T}={\left[\cos\left({\hat{\psi }}_{k}\right),\sin\left({\hat{\psi }}_{k}\right)\right]}^{⊤},\;\hat{N}={\left[-\sin\left({\hat{\psi }}_{k}\right),\cos\left({\hat{\psi }}_{k}\right)\right]}^{⊤}$$

These vectors form the local Frenet-Serret frame. All subsequent high-frequency displacement drifts and GNSS innovations are projected onto this basis to compute independent, direction-specific PLs.

### 4.2. Directional Drift and Geometric Quality

To accurately estimate the true error rate, the system monitors the direct physical discrepancies between the ES-EKF output and the raw IMU dead-reckoning. Given two successive ES-EKF positions, $p_{k-1}$ and $p_{k}$, the observed global displacement is:

(3) $$\Delta p=p_{k}-p_{k-1}$$

This is compared against the theoretical displacement dictated purely by the IMU integration:

(4) $$\Delta p_{\mathrm{dr}}=v_{k-1}\Delta t+\frac{1}{2}\left(R_{k}a_{k}-g\right)\Delta t^{2}$$

where $g={\left[0,0,9.78\right]}^{⊤}$ is the gravity vector, $R_{k}$ is the rotation matrix of the body frame to the world frame, and $a_{k}$ is the acceleration. The directional drift between these two estimates exposes unobservable integration errors:

(5) $$d=\Delta p-\Delta p_{\mathrm{dr}}$$

Projecting this slip onto the local vehicle frame yields the directional drift magnitudes:

(6) $$d_{\mathrm{long}}=|d\cdot \hat{T}|,\;d_{\mathrm{lat}}=\left|d\cdot \hat{N}\right|$$

Beyond internal directional drift, the system must evaluate its external environmental perception. The geometric quality of the LiDAR scan-to-map registration is quantified by the root mean square residual:

(7) $$r_{\mathrm{geo}}=\sqrt{\frac{1}{|V_{k}|}\sum_{i\in V_{k}}{∥p_{i}^{\mathrm{map}}-m\left(p_{i}^{\mathrm{scan}}\right)∥}_{2}}\;\left[m\right]$$

where $m:R^{3}\to R^{3}$ maps a source point to its nearest neighbor in the global map. However, a low residual $r_{\mathrm{geo}}$ does not guarantee high positional accuracy if the geometric constraints are degenerate. The mapping from residual space to state error is dictated by the Jacobian matrix J of the registration. We define the environmental sensitivity factor ξ as the minimum eigenvalue of the Hessian [31]:

(8) $$\xi =\frac{1}{\sqrt{\lambda_{\min}\left(J^{⊤}J\right)+\epsilon }}\;\left[m^{-1}\right]$$

where ϵ is a small positive number to avoid singularity. A small $\lambda_{\min}$ implies geometric degeneracy, inflating ξ to penalize any observed residual.

### 4.3. Instantaneous Error Rate

In this work, we assume that the position error evolves through three independent contributing factors, whose effects combine additively to form the instantaneous error rate. Although the independence condition may not strictly hold in practice, the sum of the individual error bounds provides a guaranteed conservative upper bound on the total error. Let $e_{1}\left(t\right)$, $e_{2}\left(t\right)$, and $e_{3}\left(t\right)\geq 0$ denote the deterministic bounds of the individual error contributions from IMU drift, directional drift, and geometric degradation, respectively. The validity of this additive combination follows from the fundamental inequality:

(9) $$e_{1}+e_{2}+e_{3}\geq \sqrt{e_{1}^{2}+e_{2}^{2}+e_{3}^{2}}$$

Squaring both sides preserves the inequality since all terms are non-negative:

(10) $$\begin{array}{cc} {\left(e_{1}+e_{2}+e_{3}\right)}^{2} & \geq e_{1}^{2}+e_{2}^{2}+e_{3}^{2} \end{array}$$

(11) $$\begin{array}{cc} e_{1}^{2}+e_{2}^{2}+e_{3}^{2}+2\left(e_{1}e_{2}+e_{1}e_{3}+e_{2}e_{3}\right) & \geq e_{1}^{2}+e_{2}^{2}+e_{3}^{2} \end{array}$$

(12) $$\begin{array}{cc} 2(e_{1}e_{2}+e_{1}e_{3}+e_{2}e_{3}) & \geq 0 \end{array}$$

Since $e_{1},e_{2},e_{3}\geq 0$, all cross-terms; indicating the cross-correlation effect, are non-negative, ensuring the inequality holds. Based on the above, for a given direction i∈{long,lat}, we define the continuous-time error rate as:

(13) $$\Gamma_{i}\left(t\right)=\underset{\mathrm{IMU}}{\underset{︸}{\sigma_{\mathrm{base}}}}+\underset{\mathrm{directional}\;\mathrm{drift}}{\underset{︸}{w_{\mathrm{drift}}\cdot \frac{|d_{i}|}{\Delta t}}}+\underset{\mathrm{Geometric}\;\mathrm{Degradation}}{\underset{︸}{w_{\mathrm{geo}}\cdot r_{\mathrm{geo}}\left(t\right)\cdot \xi \left(t\right)}}\;\left[m/s\right]$$

Here, $\sigma_{\mathrm{base}}$ [m/s] represents the irreducible baseline drift arising from the IMU velocity random walk. The directional drift $|d_{i}|$ [m] is divided by Δt to convert it to a velocity, rendering $w_{\mathrm{drift}}$ dimensionless. Since ξ has units of $\left[m^{-1}\right]$ and $r_{\mathrm{geo}}$ is expressed in [m], their product is dimensionless; consequently, $w_{\mathrm{geo}}$ [m/s] determines the rate at which geometric degeneracy translates into positional error. The weights $w_{\mathrm{drift}}$ and $w_{\mathrm{geo}}$ are conservative scaling factors selected to ensure dimensional consistency and to reflect the appropriate relative influence of each error source based on sensor characteristics and system physics.

### 4.4. Setpoint Formulation and Saturation Dynamics

The variable $\Gamma_{i}\left(t\right)$ represents a velocity; the rate at which positional error is expanding. Mathematically, to find the accumulated positional error boundary, we must integrate this rate over time (S(t)=∫Γ(t)dt). However, pure integration of a strictly positive error rate yields an unbounded function ($\lim_{t\to \infty }S\left(t\right)=\infty$). This is physically unrealistic; positional uncertainty is implicitly bounded by kinematics, prior maps, and absolute constraints. To resolve this, we introduce the *Setpoint* S(t), representing the mathematically ideal safety bound. Instead of pure integration, we model its evolution using a bounded-growth differential equation:

(14) $$\frac{dS(t)}{dt}=g\left(S\left(t\right)\right)\cdot \Gamma \left(t\right)$$

Here, g(S)∈[0,1] is a dimensionless saturation function that dampens the growth rate as the uncertainty approaches a maximum operational ceiling, $S_{\max}$:

(15) $$g\left(S\left(t\right)\right)=1-{\left(\frac{S(t)}{S_{\max}}\right)}^{n},\;n>0,\;0\leq S\leq S_{\max}$$

When the accumulated uncertainty is small ($S\left(t\right)\ll S_{\max}$), the penalty term is negligible (g(S)≈1). In this regime, $\frac{dS}{dt}\approx \Gamma \left(t\right)$, meaning the Setpoint safely absorbs the unmitigated physical error growth. As uncertainty approaches $S_{\max}$, g(S) asymptotically converges to zero, smoothly arresting the expansion. The ceiling $S_{\max}$ defines the maximum allowable PL based on operational constraints such as lane width, corridor limits, or the acceptable alarm limit. According to the American Federal Radionavigation Plan [32,33], such limits can extend up to 20 m depending on the application. $S_{\max}$ provides a physically meaningful cap that keeps the PL finite and operationally relevant. Its selection governs the trade-off between conservatism and availability, and must be tailored to the specific deployment scenario.

Discretized via Forward Euler, the directional setpoints evolve as:

(16) $$S_{i,k}=S_{i,k-1}+\left[1-{\left(\frac{S_{i,k-1}}{S_{\max}}\right)}^{n}\right]\cdot \Gamma_{i,k}\cdot \Delta t$$

When global positioning innovations $v_{\mathrm{gnss}}$ become available, we incorporate this new information to refine the setpoint. The innovation magnitude $|v_{\mathrm{gnss}}|$ represents the disagreement between the current state estimate and the GNSS observation.

To account for nominal GNSS operation, we incorporate the bound $K_{\mathrm{TIR}}\cdot \sigma_{\mathrm{gnss}}$, where $K_{\mathrm{TIR}}$ is an inflation factor that maps the TIR to a standard normal multiplier [7]. The setpoint is then updated as the minimum between its current value and this new bound:

(17) $$\begin{array}{cc} S_{\mathrm{long},k} & \leftarrow \min\left(S_{\mathrm{long},k},\left|v_{\mathrm{gnss}}\cdot \hat{T}\right|+K_{\mathrm{TIR}}\cdot \sigma_{\mathrm{gnss}}\right) \end{array}$$

(18) $$\begin{array}{cc} S_{\mathrm{lat},k} & \leftarrow \min\left(S_{\mathrm{lat},k},\left|v_{\mathrm{gnss}}\cdot \hat{N}\right|+K_{\mathrm{TIR}}\cdot \sigma_{\mathrm{gnss}}\right) \end{array}$$

This architecture explicitly decouples the verification of safety bounds from the state estimation process, rendering explicit fault detection and exclusion modules unnecessary for the PL itself.

The update rule follows directly from the triangle inequality. Let $p_{i}$ denote the true position along a projected axis, ${\hat{p}}_{i,k-1}$ the prior global state estimate, and $z_{i,k}$ the GNSS measurement. The true prior error $e_{i}^{-}=\left|{\hat{p}}_{i,k-1}-p_{i}\right|$ is bounded by the current setpoint $S_{i,k-1}$. By introducing the measurement into the error expression, we obtain $e_{i}^{-}=\left|\left({\hat{p}}_{i,k-1}-z_{i,k}\right)+\left(z_{i,k}-p_{i}\right)\right|$. Applying the triangle inequality gives:

(19) $$|{\hat{p}}_{i,k-1}-p_{i}\left|\;\leq \;\right|{\hat{p}}_{i,k-1}-z_{i,k}\left|\;+\;\right|z_{i,k}-p_{i}|$$

The first term on the right is precisely the projected innovation magnitude $|v_{\mathrm{gnss}}\cdot \hat{T}|$, while the second term, $|z_{i,k}-p_{i}|$, is bounded by $K_{\mathrm{TIR}}\sigma_{\mathrm{gnss}}$ [7]. This leads to the following inequality:

(20) $$e_{i}^{-}\leq S_{\mathrm{gnss},i}=v_{\mathrm{proj}}+K_{\mathrm{TIR}}\sigma_{\mathrm{gnss}}$$

Since the true error must satisfy both the set point *S*, prior bound and the GNSS-derived bound, it is rigorously bounded by their intersection:

(21) $$e_{i}^{-}\leq \min\left(S_{i,k-1},S_{\mathrm{gnss},i}\right)$$

This structure inherently protects against measurement faults. If a GNSS measurement is corrupted, it will diverge significantly from the prior estimate, resulting in a large innovation $|v_{\mathrm{gnss}}|$. Consequently, the candidate GNSS bound $S_{\mathrm{gnss},i}$ inflates naturally. The minimum operator then safely rejects this inflated bound, retaining the tighter prior setpoint.

If the global localization filter subsequently fuses a corrupted GNSS measurement, the posterior state estimate undergoes a spatial jump. Let $\Delta {\hat{p}}_{i}={\hat{p}}_{i,k}-{\hat{p}}_{i,k-1}$ denote this jump. The true error after fusion is bounded as:

(22) $$e_{i}^{+}\;=\;|{\hat{p}}_{i,k}-p_{i}\left|\;=\;\right|{\hat{p}}_{i,k-1}+\Delta {\hat{p}}_{i}-p_{i}\left|\;\leq \;\right|{\hat{p}}_{i,k-1}-p_{i}|\;+\;|\Delta {\hat{p}}_{i}|$$

Since the prior error is bounded by the setpoint $|{\hat{p}}_{i,k-1}-p_{i}|\leq S_{i,k-1}$, we obtain:

(23) $$e_{i}^{+}\leq S_{i,k-1}+\left|\Delta {\hat{p}}_{i}\right|$$

At this instant, the PL remains at $S_{i,k-1}$ and has not yet expanded to accommodate the injected error. However, in the subsequent epoch, the directional drift, $d=\Delta p-\Delta p_{\mathrm{dr}}$, detects this jump. Because the physical IMU integration $\Delta p_{\mathrm{dr}}$ remains continuous and reflects only nominal motion, while the filter displacement Δp includes the artificial jump, the drift magnitude becomes:

(24) $$|d|\approx |\Delta {\hat{p}}_{i}|$$

This spike in the drift magnitude $|d_{i}|$ immediately drives the error rate $\Gamma_{i}\left(t\right)$ upward. With $w_{\mathrm{drift}}=1$, for simplicity, the setpoint expands in the next epoch as:

(25) $$S_{i,k+1}=S_{i,k}+\Gamma_{i}\Delta t\geq S_{i,k-1}+\left|\Delta {\hat{p}}_{i}\right|+\sigma_{\mathrm{base}}\Delta t$$

The geometric term $w_{\mathrm{geo}}\cdot r_{\mathrm{geo}}\cdot \xi$ is omitted here as it would only add additional expansion; the inequality represents a conservative lower bound. Thus, by the next epoch, the PL has expanded by at least the magnitude of the injected fault. Combining the two inequalities yields:

(26) $$e_{i}^{+}\leq S_{i,k-1}+\left|\Delta {\hat{p}}_{i}\right|\leq S_{i,k+1}$$

The one-epoch delay is bounded by the sampling period Δt, which is orders of magnitude smaller than the vehicle’s dynamic response time, ensuring that the PL expands before the error can cause unsafe operation.

### 4.5. Protection Level Tracking via Gain-Scheduling PID Control

To prevent the aforementioned discontinuity, we deploy a Proportional-Integral-Derivative (PID) controller. The PID controller acts as a dynamic shock absorber. Its purpose is to track the highly volatile setpoint, $S_{k}$, and generate a smooth velocity command, $u_{i,k}$, that drives the operational safety bound, $PL_{k}$, toward the target without aggressive jumps. The controller minimizes the tracking error $e_{i,k}$, defined as the gap between where the safety bound *should* be, $S_{i,k}$, and where it currently *is*, $PL_{i,k-1}$:

(27) $$e_{i,k}=S_{i,k}-PL_{i,k-1}$$

#### 4.5.1. Shannon Entropy and Gain Scheduling

A static PID controller will underperform as the vehicle transitions between different environments. Therefore, we adapt the PID gains dynamically based on the *Shannon Entropy* of the local environment. The Shannon entropy, $H_{k}$, quantifies the chaos or uncertainty in the LiDAR registration. We compile the LiDAR ICP [34] point-to-map residuals into a normalized histogram with $N_{\mathrm{bins}}$ bins, approximating a discrete probability distribution P(j) over residual values. The normalized Shannon entropy is then:

(28) $$H_{k}=\frac{-\sum_{j}P\left(j\right)\log_{2}P\left(j\right)}{\log_{2}\left(N_{\mathrm{bins}}\right)}\;\in \left[0,1\right]$$

The denominator $\log_{2}\left(N_{\mathrm{bins}}\right)$ normalizes the entropy to [0,1], where values near 0 indicate highly concentrated residuals (predictable, low-entropy environments like flat walls) and values near 1 indicate widely dispersed residuals (chaotic, high-entropy environments like a cluttered environment with pedestrians). We use $H_{k}$ to adapt the nominal base gains ($K_{p}^{0},K_{i}^{0},K_{d}^{0}$) according to the following linear scaling laws:

(29) $$\begin{array}{cc} K_{p}\left(H_{k}\right) & =K_{p}^{0}\left(1+\alpha_{p}H_{k}\right) \end{array}$$

(30) $$\begin{array}{cc} K_{i}\left(H_{k}\right) & =K_{i}^{0}\left(1-\alpha_{i}H_{k}\right) \end{array}$$

(31) $$\begin{array}{cc} K_{d}\left(H_{k}\right) & =K_{d}^{0}\left(1-\alpha_{d}H_{k}\right) \end{array}$$

The scheduling laws are governed by positive scaling coefficients $\alpha_{p}$, $\alpha_{i}$, and $\alpha_{d}$, with a *linear mapping* chosen because the *environmental entropy $H_{k}$ is strictly bounded between 0 and 1*. This ensures that the controller gains scale smoothly and proportionally to the level of environmental degradation. As $H_{k}\to 1$, the proportional gain $K_{p}$ increases by $1+\alpha_{p}H_{k}$ to drive *rapid* expansion of the PL in response to growing uncertainty. Conversely, the integral gain $K_{i}$ decreases by $1-\alpha_{i}H_{k}$ to *prevent* accumulation of noisy measurement spikes, allowing the PL to recover when conditions improve. Similarly, the derivative gain $K_{d}$ decreases by $1-\alpha_{d}H_{k}$ to *desensitize* the controller to high-frequency noise. This dynamic scheduling enforces aggressive threat response while preventing error accumulation and noise amplification.

#### 4.5.2. PID Components

The final control velocity $u_{i,k}$ [m/s] is generated by summing three components [35]:

(32) $$u_{i,k}=\underset{\mathrm{Proportional}}{\underset{︸}{K_{p}\left(H_{k}\right)e_{i,k}}}+\underset{\mathrm{Integral}}{\underset{︸}{K_{i}\left(H_{k}\right)I_{i,k}}}+\underset{\mathrm{Derivative}}{\underset{︸}{K_{d}\left(H_{k}\right)D_{i,k}}}$$

*1. The Integral Accumulator ($I_{i,k}$):* Introduced to eliminate steady-state error, $I_{i,k}$ accumulates the tracking error over time so that the PL eventually reaches the setpoint, even when the proportional term alone is insufficient. However, if the PL hits its absolute physical ceiling, $S_{\max}$, and cannot grow further, a standard integrator would continue accumulating error indefinitely; a failure known as “integral windup.” To prevent this, we implement a *Conditional Integration Anti-Windup* scheme [36]:

(33) $$I_{i,k}=\left\{\begin{array}{cc} I_{i,k-1}+e_{i,k}\Delta t, & \mathrm{if}\;|I_{i,k-1}|\;<\gamma S_{\max}\;\mathrm{or}\;\left(e_{i,k}\cdot I_{i,k-1}<0\right)\\ I_{i,k-1}, & \mathrm{otherwise}\;(\mathrm{windup}\;\mathrm{detected}) \end{array}\right.$$

The threshold γ∈(0,1) defines the maximum allowable integral magnitude relative to the physical ceiling $S_{\max}$.

*2. The Filtered Derivative ($D_{i,k}$):* When the setpoint $S_{k}$ changes abruptly—for instance, after a GNSS update—the tracking error $e_{i,k}$ becomes large in magnitude. The proportional term alone would command an aggressive correction. The derivative term detects this rapid change and provides damping, ensuring the PL transitions smoothly toward the new setpoint without overshoot or oscillation. We pass the derivative through a first-order low-pass filter [37]:

(34) $$\begin{array}{cc} \alpha_{\mathrm{filt}} & =\min\left(1,\;2\pi f_{c}\Delta t\right) \end{array}$$

(35) $$\begin{array}{cc} D_{i,k} & =\alpha_{\mathrm{filt}}\left(\frac{e_{i,k}-e_{i,k-1}}{\Delta t}\right)+\left(1-\alpha_{\mathrm{filt}}\right)D_{i,k-1} \end{array}$$

The cutoff frequency $f_{c}$ determines the filter’s bandwidth. The coefficient $\alpha_{\mathrm{filt}}\in \left(0,1\right]$ controls how much weight is given to the current raw derivative versus the previous filtered value.

### 4.6. Asymmetric Safety Update and Bounds Enforcement

The PID controller output $u_{i,k}$ is a velocity command [m/s] dictating how fast the PL should move. To update the actual positional boundary [m], we integrate this velocity over the discrete timestep Δt. We enforce asymmetric dynamics to reflect a conservative safety philosophy: uncertainty increases must be respected immediately, while uncertainty decreases should be treated with skepticism. A decay modifier $\alpha_{\mathrm{decay}}\in \left(0,1\right)$ governs this behavior:

(36) $$PL_{i,k}=PL_{i,k-1}+\left\{\begin{array}{cc} u_{i,k}\Delta t, & \mathrm{if}\;u_{i,k}\geq 0\;(\mathrm{rising}:\;\mathrm{full}\;\mathrm{speed})\\ \alpha_{\mathrm{decay}}\cdot u_{i,k}\Delta t, & \mathrm{if}\;u_{i,k}<0\;(\mathrm{falling}:\;\mathrm{damped}) \end{array}\right.$$

When the controller commands expansion, $u_{i,k}\geq 0$, the safety bound increases immediately at full speed. When it commands contraction, $u_{i,k}<0$, the descent is throttled by $\alpha_{\mathrm{decay}}$, requiring sustained evidence of safety before the bound relaxes.

Finally, the updated PL is clamped to absolute bounds, preventing it from falling below the minimum error from base IMU drift or exceeding the operational ceiling $S_{\max}$:

(37) $$PL_{i,k}=\max\left(K_{\mathrm{TIR}}\cdot \sigma_{\mathrm{base}}\cdot \Delta t,\;\min\left(PL_{i,k},S_{\max}\right)\right)$$

This formula serves as a safety-critical bounding mechanism. The max(·) operator enforces the lower bound, representing the irreducible sensor noise floor. Even in perfect environments, the system uncertainty cannot vanish due to inherent sensor noise; primarily the IMU noise, characterized by $\sigma_{\mathrm{base}}$ inflated by $K_{\mathrm{TIR}}$ [7,8]. The min(·) operator enforces the upper bound, corresponding to the absolute alarm limit represented by $S_{\max}$. Exceeding this limit indicates an unsafe operational state. Tracking a PL beyond $S_{\max}$ is therefore operationally meaningless. Moreover, pinning the PL at $S_{\max}$ during prolonged degradation triggers the controller’s anti-windup logic, preventing unbounded integral accumulation and ensuring rapid recovery once conditions improve.

The complete algorithmical flow of this method is detailed in Algorithm A1.

### 4.7. Smoothness and Stability

We establish that the PL sequence $\left\{PL_{i,k}\right\}$ evolves smoothly between consecutive time steps and that all operational variables remain strictly bounded within a valid physical domain. The sequence $\left\{PL_{i,k}\right\}$ per-step variation is strictly governed by the sampling period Δt. The PID velocity command is given by Equation (32), where each term in the velocity command is bounded:

- The gain-scheduling functions are bounded because environmental entropy is normalized ($H_{k}\in \left[0,1\right]$).
- The tracking error $e_{i,k}=S_{i,k}-PL_{i,k-1}$ is bounded because both the setpoint $S_{i,k}$ and PL $PL_{i,k}$ are bounded by $S_{\max}$.
- The term $I_{i,k}$ is bounded by the anti-windup condition ($|I_{i,k}|\leq \gamma S_{\max}$).
- The term $D_{i,k}$ is bounded as the output of a stable first-order low-pass filter driven by bounded tracking errors.

Consequently, the control command is strictly upper-bounded by a finite constant, $|u_{i,k}|\;\leq u_{\max}$, for all k≥0.

The PL update, Equation (36), can be written:

(38) $${PL}_{i,k}={PL}_{i,k-1}+\Delta {PL}_{i,k},\;\Delta {PL}_{i,k}=h\left(u_{i,k}\right)\Delta t$$

where the asymmetric rate function h(u) is defined as:

(39) $$h\left(u\right)=\left\{\begin{array}{cc} u, & u\geq 0\\ \alpha_{\mathrm{decay}}\cdot u, & u<0 \end{array}\right.$$

The function h(u) is continuous everywhere on R, satisfying $\lim_{u\to 0^{+}}h\left(u\right)=\lim_{u\to 0^{-}}h\left(u\right)=0$. Since $0<\alpha_{\mathrm{decay}}<1$, the per-epoch change in PL is strictly bounded by:

(40) $$|\Delta PL_{i,k}|\;=|h\left(u_{i,k}\right)|\Delta t\leq u_{\max}\Delta t$$

Because the step magnitude $|\Delta {PL}_{i,k}|$ scales directly with the sampling interval Δt, the safety bound evolves smoothly without abrupt jumps, providing a well-behaved boundary for downstream tasks.

Stability follows from the characteristic equation of the closed-loop system. Because the asymmetric update, Equation (36), applies a strictly positive scaling factor (α>0), the effective gains ${\tilde{K}}_{p},{\tilde{K}}_{d},{\tilde{K}}_{i}$ remain strictly positive. The characteristic equation is given by:

(41) $$s^{2}+\left({\tilde{K}}_{p}+{\tilde{K}}_{d}\right)s+{\tilde{K}}_{i}=0$$

Since all effective gains are strictly positive, the polynomial coefficients remain positive, guaranteeing closed-loop stability [35,38].

## 5. Experimental Evaluations

**Figure 2:**
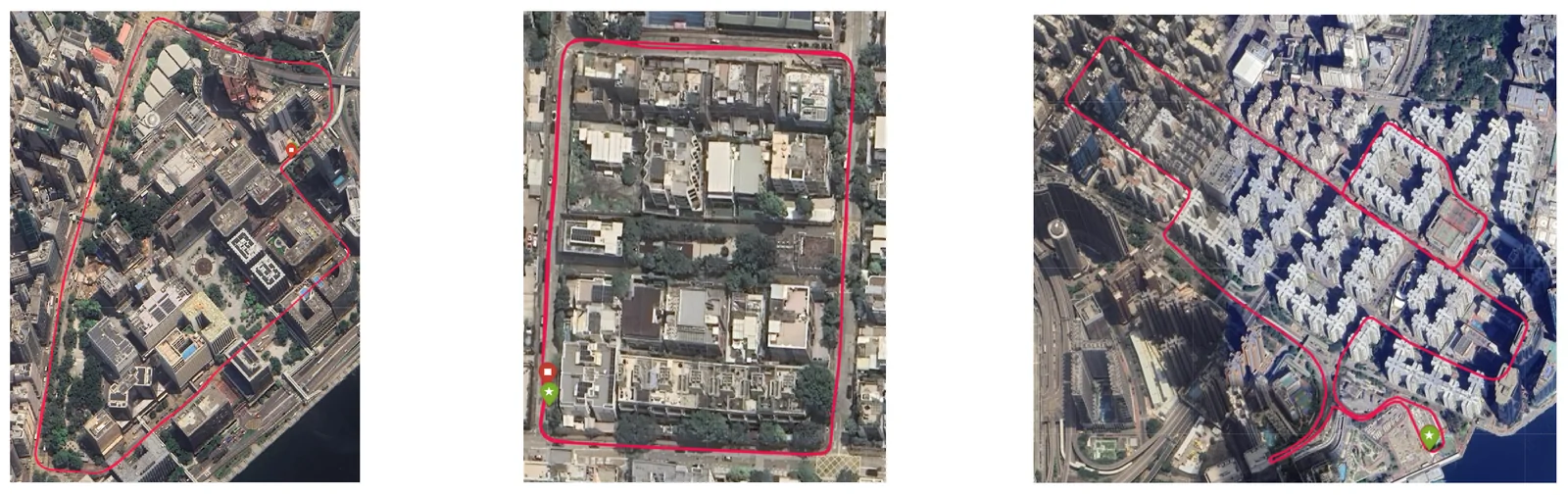
UrbanNav trajectories: (**left**) Medium urban (3.64km); (**center**) Low urban (1.21km); (**right**) Deep urban (4.51km).

We evaluate our approach on three sequences from the UrbanNavDataset [39]: medium-urban, deep-urban, and low-urban residential (see Figure 2). Performance is benchmarked against the method proposed in [11]. For each sequence, we present a time-evolution plot showing the true error alongside the computed PL. To provide a comprehensive quantitative assessment, we employ two complementary evaluation tools: the IR metric and the Stanford diagram. The Stanford diagram is a scatter plot that compares the true error against the PL, with the diagonal line representing the condition where PL equals true error. Points above this diagonal indicate safe operating conditions (the PL conservatively bounds the actual error), while points below the diagonal represent risk conditions (the actual error exceeds the PL, violating the integrity guarantee).

The following parameters were used throughout all experiments: base IMU drift rate $\sigma_{\mathrm{base}}=0.05$ m/s, $w_{\mathrm{drift}}=1.5$, $w_{\mathrm{geometric}}=0.5$, TIR $=10^{-3}$, maximum PL $S_{\max}=10.0$ m, saturation exponent n=2, nominal PID gains $K_{p}^{0}=0.2$, $K_{i}^{0}=0.05$, $K_{d}^{0}=0.001$, decay modifier $\alpha_{\mathrm{decay}}=0.15$, derivative cutoff $f_{c}=5.0$ Hz, integral saturation threshold γ=0.3, and entropy adaptation coefficients $\alpha_{p}=2.0$, $\alpha_{i}=0.8$, $\alpha_{d}=0.5$.

The weights $w_{\mathrm{drift}}$ and $w_{\mathrm{geometric}}$ values ensure dimensional consistency while emphasizing that directional drift, which directly captures sensor disagreement, contributes more than LiDAR map-matching residuals. The nominal PID gains together with the entropy adaptation coefficients yield at maximum entropy ($H_{k}=1$) a 130% increase in proportional gain for rapid threat response, an 80% reduction in integral gain to prevent error accumulation, and a 50% reduction in derivative gain to suppress noise amplification. *Scheduling reflects our objective of overbounding and smoothing rather than achieving a specific classical control performance specification*. The decay modifier $\alpha_{\mathrm{decay}}$ value enforces asymmetric dynamics: full-speed rise, slow decay. The integral saturation threshold γ value limits the integral contribution to 30% of the operational ceiling, preventing windup. *All parameters were fixed prior to experimentation and applied uniformly across all three UrbanNavDataset sequences*.

### 5.1. Benchmark Methodology

We compare our method against the state-of-the-art heavy-tailed PL approach proposed by Al Hage et al. [11]. This baseline models the positional components of the covariance matrix, $P_{g}$, using a multivariate Student-*t* distribution with a fixed degree of freedom (dof) tuned manually offline. We report results for dof∈{4,5,6,7,8,9,10,100}. The baseline extracts PLs from the maximum eigenvalue of $P_{g}$. Leveraging the relationship between the Student-*t* shape matrix and the covariance matrix, the PL for a TIR is:

$$\mathrm{PL}=K_{\mathrm{TIR}}\left(dof\right)\sqrt{\left(dof-2\right)\cdot \max(\mathrm{eig}\left(P_{g}\right))}$$

where $K_{\mathrm{TIR}}\left(dof\right)$ is the inflation multiplier obtained from the cumulative density function of a 2D multivariate Student-*t* distribution.

Let $\lambda_{1},\lambda_{2}$ denote the eigenvalues of $P_{g}$ with corresponding eigenvectors $V_{1},V_{2}$. These eigenvectors are projected onto the vehicle’s along-track (at) and cross-track (ct) axes. The directional PL for axis *i* is computed by taking the maximum projected variance component:

$${\mathrm{PL}}_{i}=K_{\mathrm{TIR}}\left(dof\right)\sqrt{\left(dof-2\right)\cdot \max\left(\lambda_{1}|V_{1,i}|,\lambda_{2}\left|V_{2,i}\right|\right)}$$

### 5.2. Scenario 1: Medium Urban Environment

**Figure 3:**
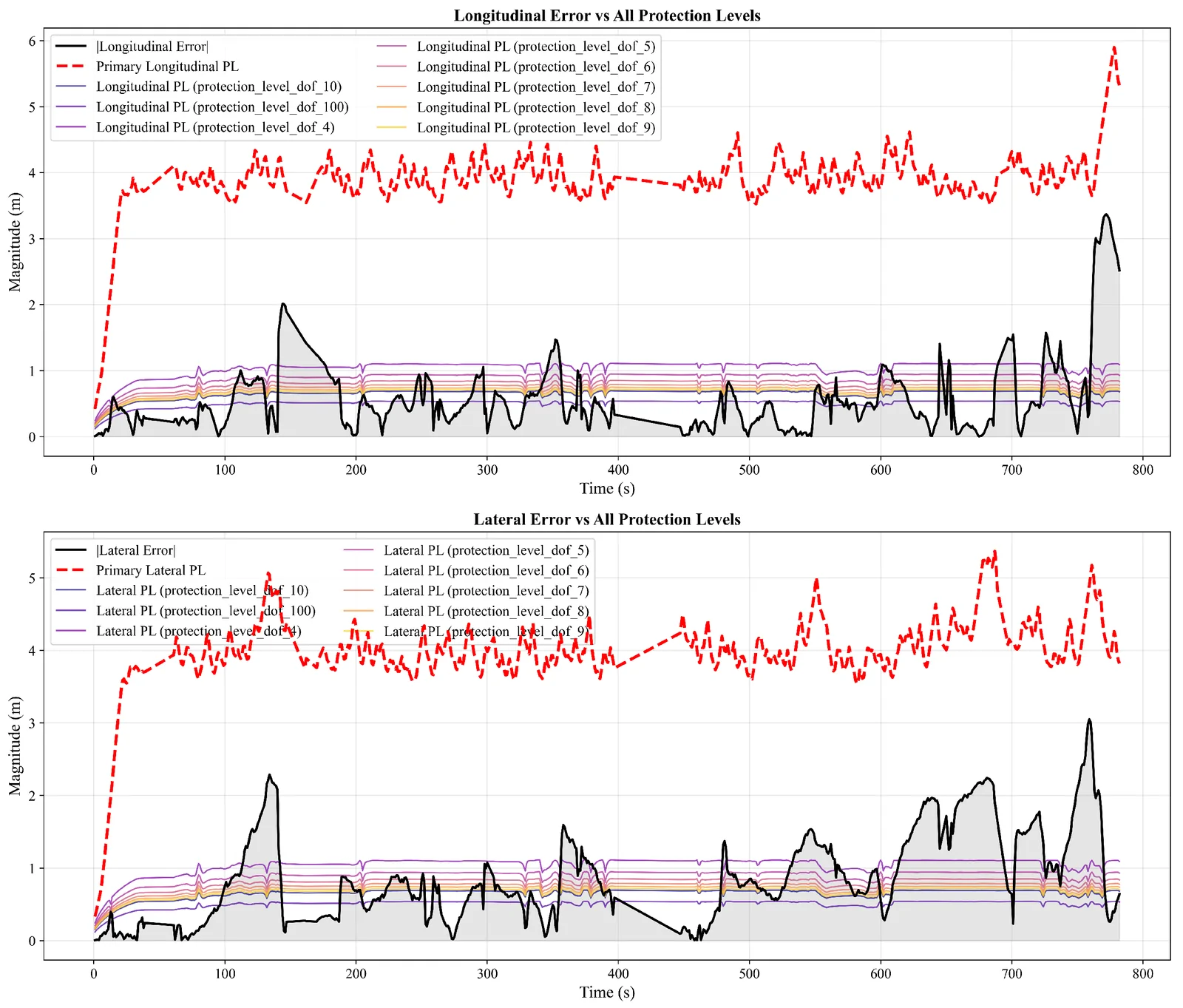
Medium-urban scenario: true error (black solid) versus proposed PID (no integral term) method (red dashed) and benchmark methods (thin solids).

The true error, Figure 3, exhibits sudden increases around 150 s and a severe surge near 750 s. These events arise from LiDAR scan-map matching degeneracy due to limited local map constraints, combined with the inherent drift of inertial propagation. The benchmark methods produce PLs that remain largely flat and do not reflect these error. Even the most conservative baseline, dof=4, remains nearly flat, largely unresponsive to actual sensor degradation. The true error reaches 3.0 m, exceeding every baseline PL.

**Figure 4:**
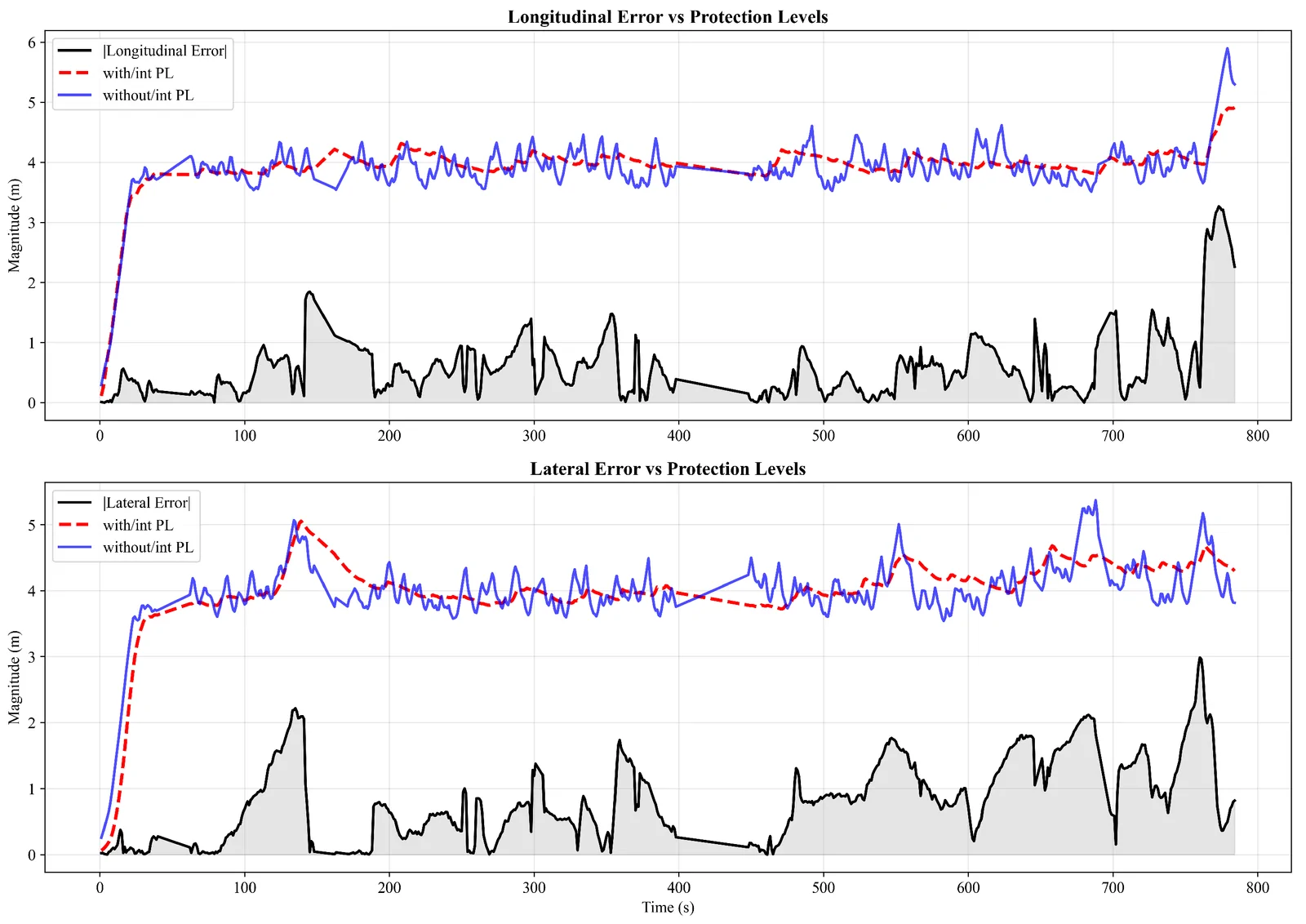
Medium-urban scenario: PL with integral term (red dashed) versus without integral term (blue solid).

The proposed method detects environmental degradation by monitoring the directional drift $d_{i}$ and the geometric sensitivity ξ through the error rate Γ(t). When geometric degeneracy occurs, $\lambda_{\min}$ decreases, inflating ξ and amplifying the contribution of the geometric residual to Γ(t), which causes the safety boundary to expand appropriately. The architectural configuration of the tracking controller significantly impacts the quality of this boundary. A formulation lacking the integral term, Figure 3, successfully overbounds the error but suffers from high-frequency oscillatory chattering, visible in the time-series evolution. Incorporating the integral term, Figure 4, absorbs steady-state offsets and dampens transient noise. The full PID framework tracks the error with a tight, smooth overbounding.

**Figure 5:**
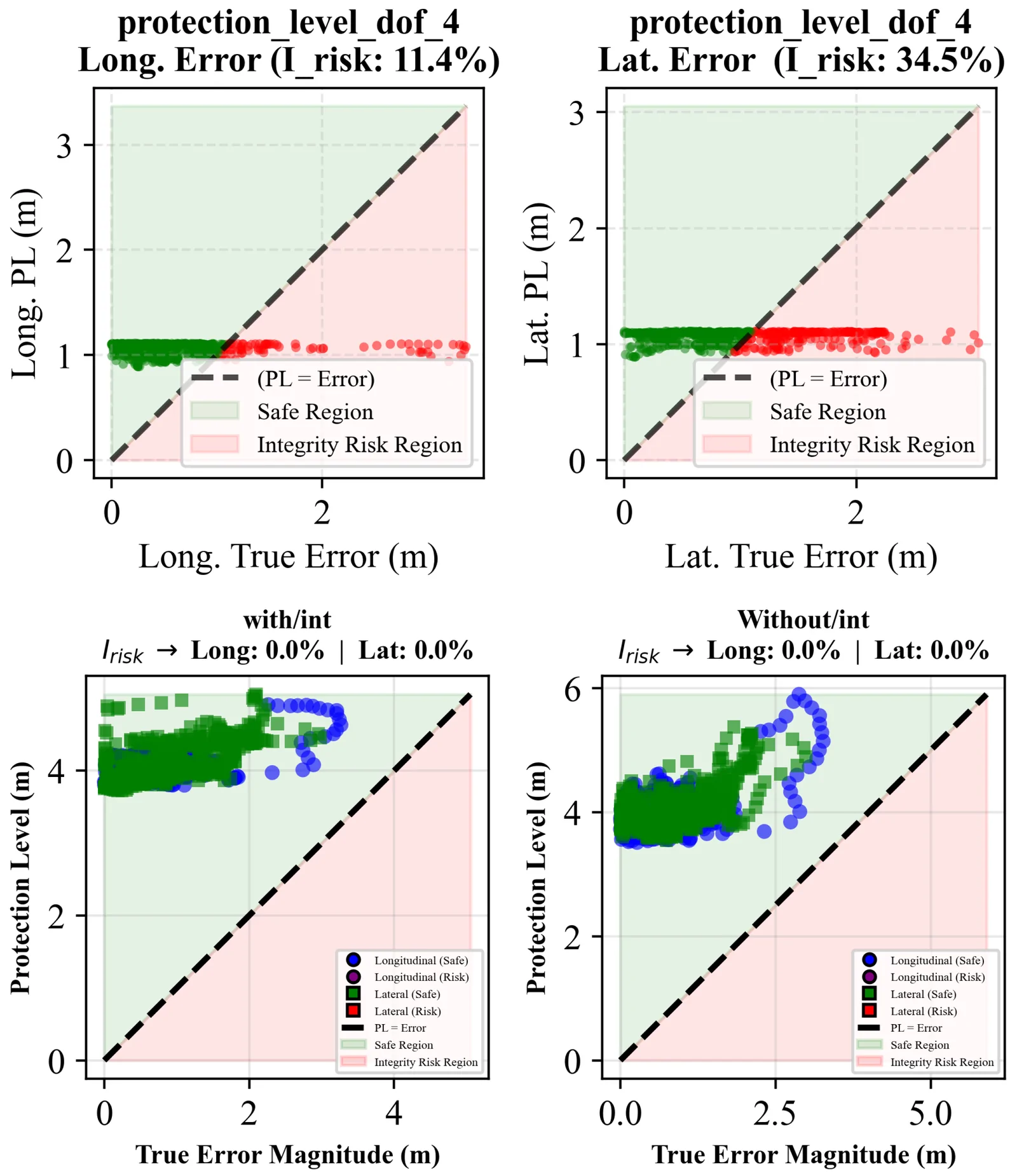
Stanford diagrams for the medium-urban scenario: (**top**) benchmark method; (**bottom**) comparison of full PID formulation (with integral) versus partial formulation (without integral).

The Stanford diagrams, Figure 5, further validate the necessity of the full PID control law. The benchmark methods exhibit limited responsiveness, with longitudinal PL reaching 11.4% and lateral risk reaching 34.5%, as their covariance matrices converge to small values and no longer reflect the true error magnitude. Both the non-integral and full PID formulations achieve 0.0% PL in both directions, safely residing in the green region with no points crossing the diagonal. However, the non-integral formulation produces a vertically dispersed scatter, indicating oscillatory behavior in the PL. The full PID method yields a significantly denser, more horizontally stable distribution, reflecting smoother and more consistent tracking performance. The resulting PL remains conservative, smooth, and free of erratic oscillations, providing a well-behaved signal for downstream planning and control modules.

### 5.3. Scenario 2: Low Urban Environment

**Figure 6:**
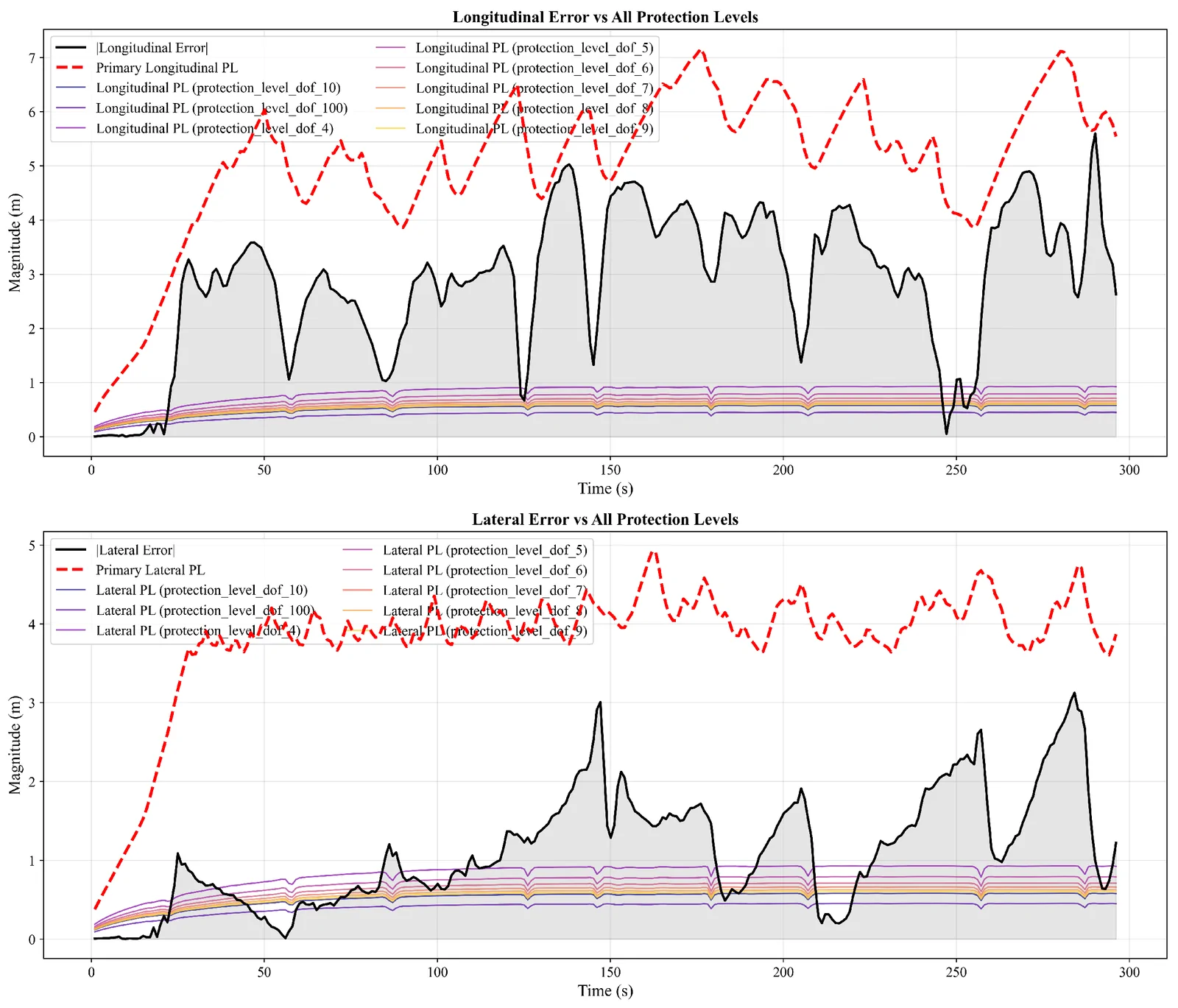
Low-urban scenario: true error (black solid) versus proposed PID (no integral term) method (red dashed) and benchmarks (thin solids).

We further evaluated the proposed framework in a low-urban environment characterized by prolonged GNSS multipath, frequent occlusions, and sparse geometric features. Unlike the previous scenario, this environment produces sustained rather than transient degradation. Figure 6 presents the time-domain evolution for this scenario.

The true error exhibits sustained volatility rather than isolated spikes, fluctuating persistently between 1 and 6 m. This indicates continuous sensor disagreement and a lack of absolute positioning references. The benchmark methods produce PLs that remain largely flat below 1 m, despite true errors reaching 6 m. Our proposed method responds robustly. The instantaneous error rate Γ(t) incorporates both the directional drift $d_{i}$ and the geometric sensitivity ξ. In this low-urban environment, the vehicle experiences prolonged GNSS degradation and frequent occlusions, causing the ES-EKF to rely more heavily on inertial propagation; thus increasing $d_{i}$ and directly inflating Γ(t). The geometric degeneracy occurs when the vehicle moves through narrow streets flanked by buildings, causing the point-cloud registration to lose constraint along certain directions. This leads to a decrease in $\lambda_{\min}\left(J^{⊤}J\right)$, which inflates the sensitivity factor ξ and amplifies the contribution of the geometric residual $r_{\mathrm{geometric}}$ to Γ(t). Simultaneously, the entropy $H_{k}$ computed from the residual distribution increases, triggering higher proportional gains for rapid response while suppressing integral and derivative gains.

**Figure 7:**
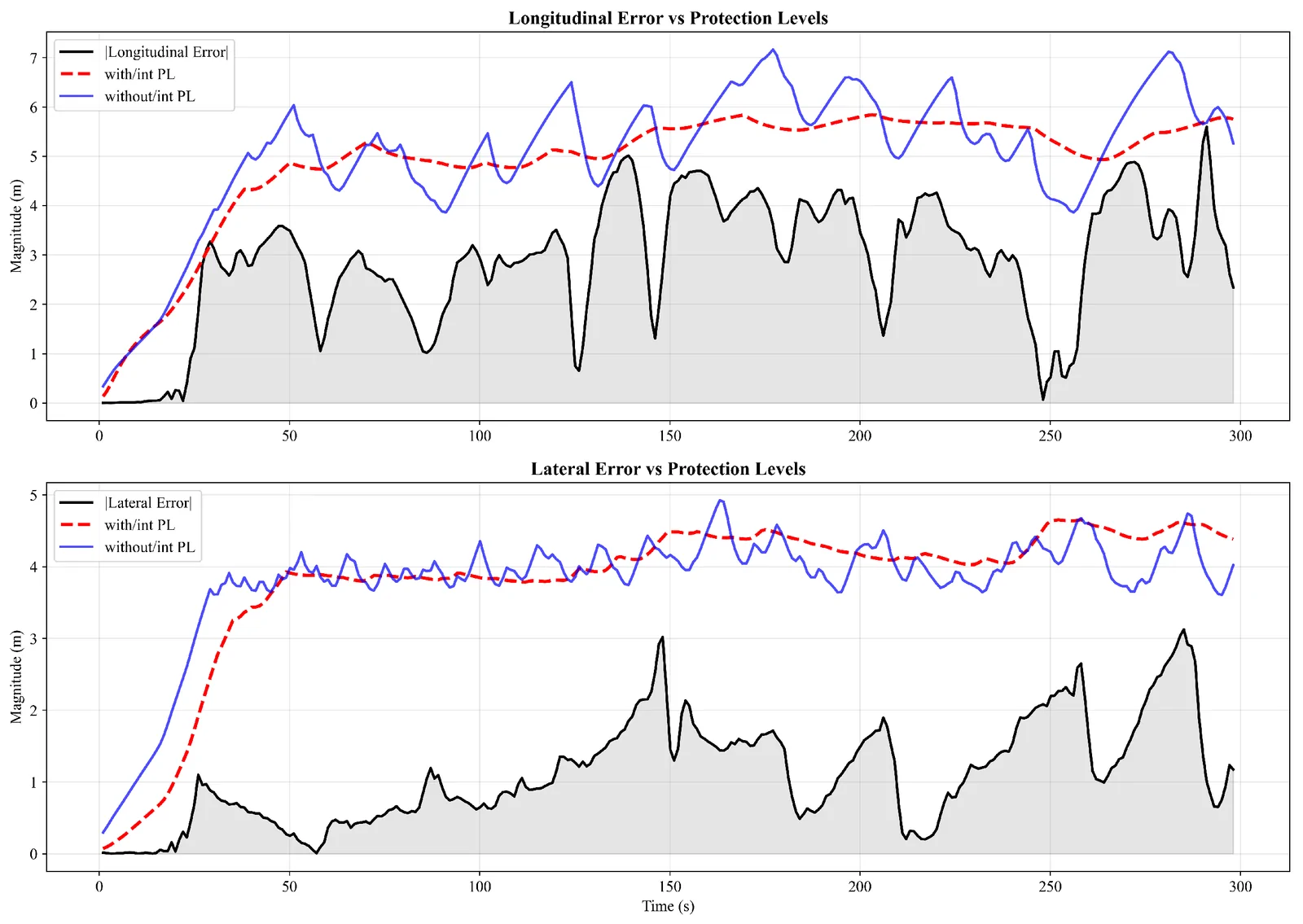
Low-urban scenario: PL with integral term (red dashed) versus without integral term (blue solid).

To isolate the impact of the controller’s architecture in managing this sustained degradation, we compared the full PID formulation against a non-integral variant, as shown in Figure 7. The non-integral formulation tracks the volatility, resulting in boundary fluctuations. By incorporating the integral term, the system effectively accumulates the steady-state error offsets inherent to sustained GNSS denial.

**Figure 8:**
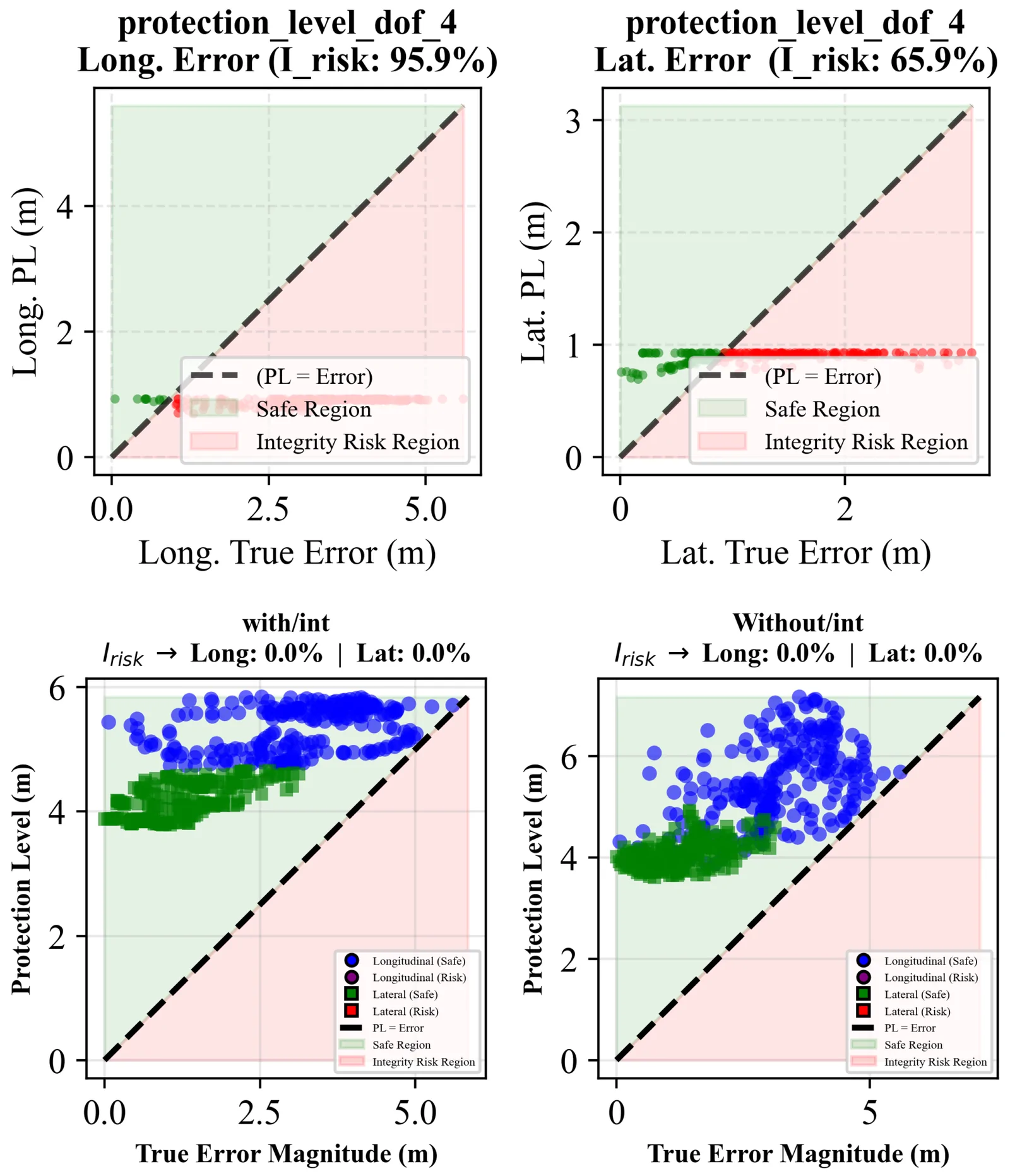
Stanford diagrams for the low-urban scenario: (**top**) benchmark method; (**bottom**) comparison of full PID formulation (with integral) versus partial formulation (without integral).

The Stanford diagrams, Figure 8, highlight the limitations of static covariance estimates in degraded environments. The baseline method yields a 95.9% PL longitudinally and 65.9% laterally. The scatter plot clusters horizontally because the PL remains near 1 m regardless of the true error magnitude.

Conversely, our dynamic control framework maintains 0.0% PL in both directions. The structural advantage of the integral component is further validated in the comparative Stanford diagrams shown in Figure 8. While both the integral and non-integral formulations successfully prevent any points from crossing into the danger region, their operational behaviors differ significantly. The non-integral method produces a widely dispersed, vertical scatter due to its oscillatory responses to sustained environmental noise.

### 5.4. Scenario 3: Deep Urban Environment

The final scenario evaluates the framework in a deep urban environment where nearly 70% of the sky-view is obstructed by buildings, causing significant GNSS degradation. This represents worst-case conditions: prolonged GNSS denial, severe multipath, and dynamic traffic.

**Figure 9:**
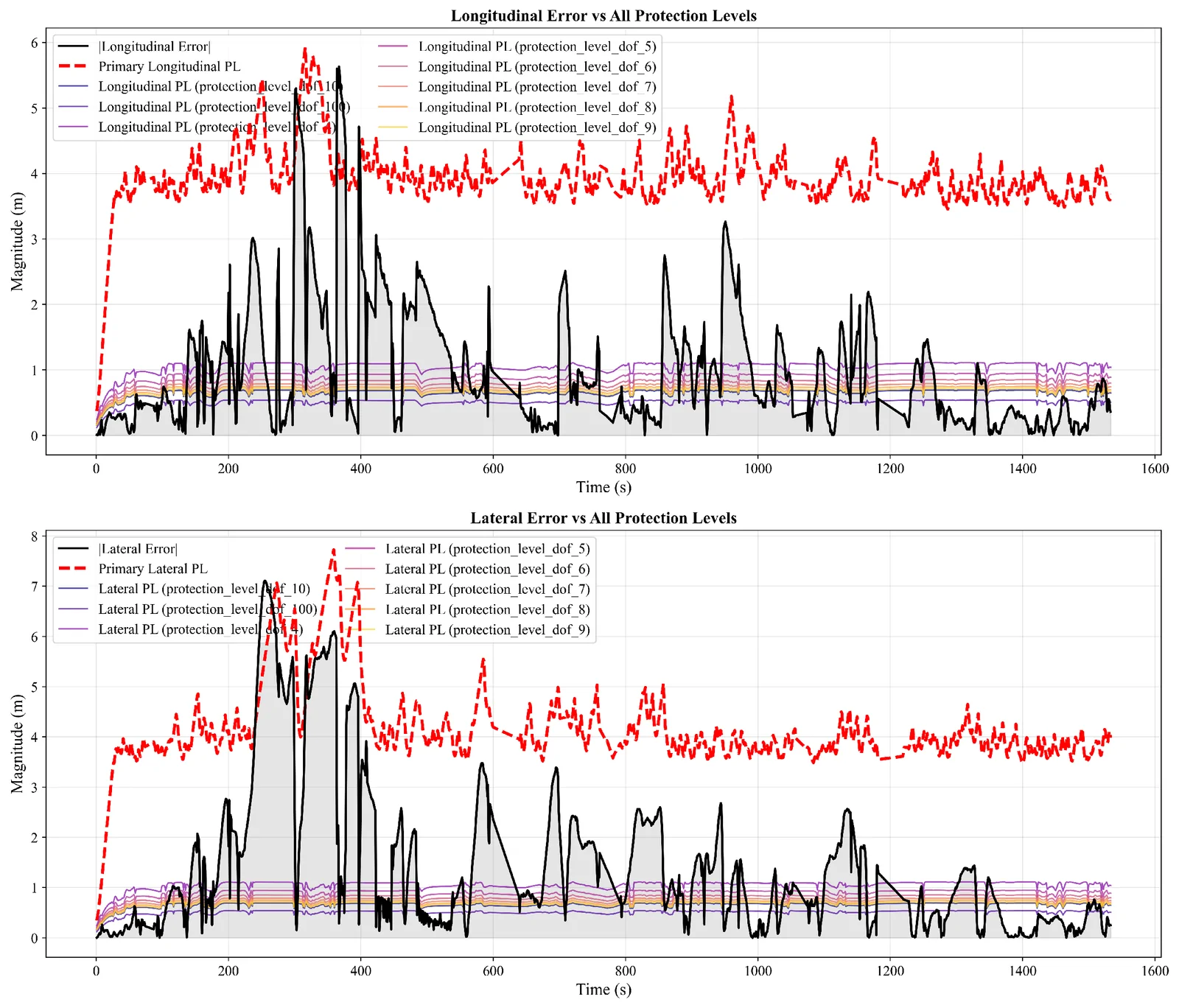
Deep urban scenario: true error (black solid) versus proposed PID (no integral term) method (red dashed) and benchmarks (thin solids).

**Figure 10:**
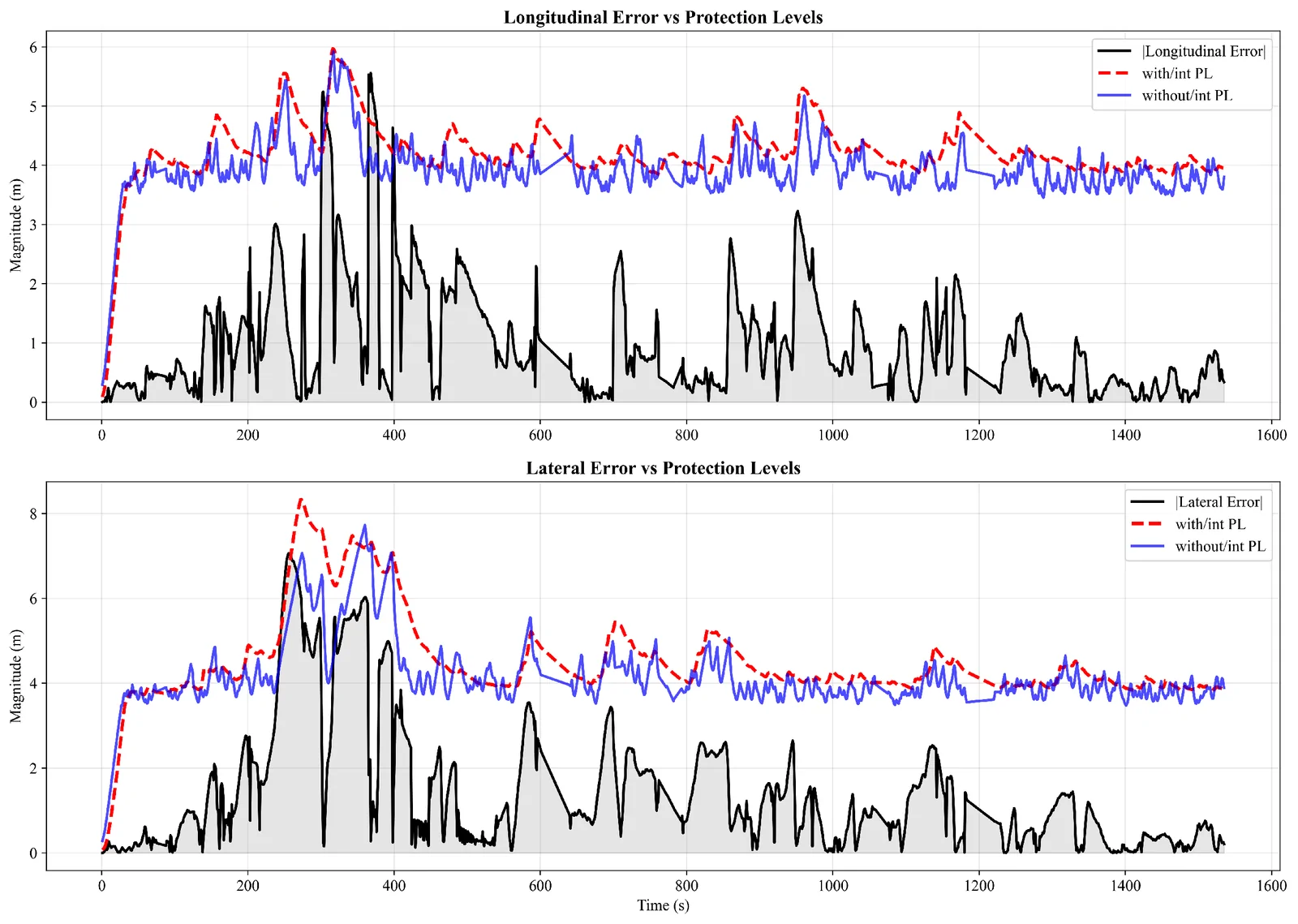
Deep-urban scenario: PL with integral term (red dashed) versus without integral term (blue solid).

The true error, Figure 9, experiences significant volatility, especially between 200 and 400 s where lateral error exceeds 7 m. This indicates near-total loss of absolute positioning constraints. The benchmark methods produce PLs that remain near 1 m, largely unresponsive to the actual true error. In this deep-urban environment, the vehicle faces multiple simultaneous challenges: degraded GNSS due to poor sky-view (nearly 70% obstruction), geometrically uninformative features that reduce the eigenvalue of $J^{⊤}J$, and dynamic objects that produce widely dispersed registration residuals. The degraded GNSS removes absolute position updates, while the feature-sparse environment drives $\lambda_{\min}$ toward minimal values, substantially inflating the sensitivity factor ξ and amplifying the geometric residual’s contribution to Γ(t). This map-matching degradation produces a two-folded effect on the tightly coupled ES-EKF: it amplifies $r_{\mathrm{geometric}}$ via the inflated ξ, while simultaneously increasing the directional drift $d_{i}$ as the filter relies more heavily on inertial propagation. These mutually reinforcing effects drive the true error between 200 and 400 s, where lateral error exceeds 7 m. Dynamic objects further increase the entropy $H_{k}$, triggering aggressive proportional gains while suppressing integral and derivative terms. Figure 10 illustrate the effect of the integral term as explained previously.

Our proposed method responds actively. By integrating directional drift and the sensitivity-weighted geometric residual, the PID controller expands the safety boundary and successfully bounds most of the 1600 s run. However, at the peak of degradation near t≈300 s, the true error gradient is sufficiently steep that it briefly exceeds the deployed PL, revealing the fundamental limit of relative localization when absolute positioning is unavailable and geometric constraints are weak.

**Figure 11:**
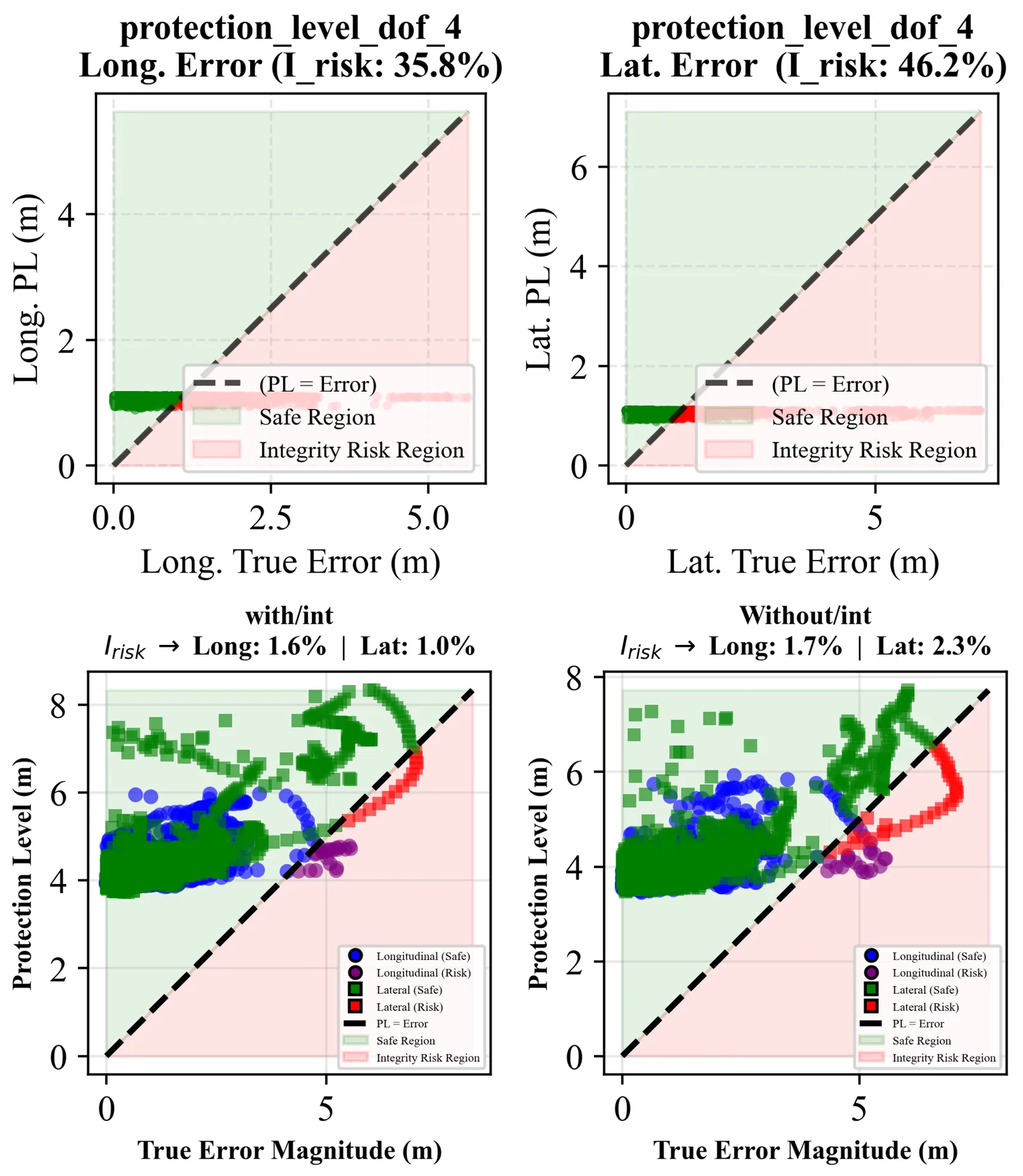
Stanford diagrams for the deep-urban scenario: (**top**) benchmark method; (**bottom**) comparison of full PID formulation (with integral) versus partial formulation (without integral).

The Stanford diagrams, Figure 11, reveal the limitations of static covariance estimation in deep urban environments. The baseline method yields 35.8% longitudinal and 46.2% lateral PL, indicating that the PLs would underestimate the true error nearly half the time under dense urban traffic conditions. Our proposed framework shows graceful degradation. Unlike previous scenarios that achieved 0.0% risk, the deep urban environment pushes the system to its limits, producing 1.7% longitudinal and 2.3% lateral PL non-integral PL and 1.6% longitudinal and 1.0% lateral with integral, with a small number of points crossing the diagonal. This minor breach is scientifically informative. It demonstrates a fundamental limit of dead-reckoning and relative geometric localization: when an environment lacks distinctive features and absolute positioning is unavailable for extended periods, no purely relative bounding algorithm can guarantee absolute safety. Nevertheless, our dynamic control framework reduces PL from 46.2% to 1.0%. By decoupling target bounds and explicitly modeling the instantaneous error rate, the PID framework degrades gracefully under extreme stress. The PL remains smooth throughout most of the trajectory, providing a well-behaved signal for downstream planners. This represents a substantial improvement in safety compared to traditional covariance-based approaches.

To address the minor integrity breach observed in the deep-urban scenario, we investigate the effect of the saturation ceiling $S_{\max}$ on the system’s ability to track extreme error. The saturation function $g\left(S\left(t\right)\right)=1-{\left(S\left(t\right)/S_{\max}\right)}^{n}$ governs the evolution of the setpoint S(t) by modulating the error rate Γ(t). As S(t) approaches $S_{\max}$, the sensitivity of the growth rate to these weights diminishes. Consider the partial derivatives:

(42) $$\frac{\partial \dot{S}}{\partial w_{\mathrm{drift}}}=g\left(S\left(t\right)\right)\cdot \frac{|d_{i}|}{\Delta t},\;\frac{\partial \dot{S}}{\partial w_{\mathrm{geo}}}=g\left(S\left(t\right)\right)\cdot r_{\mathrm{geo}}\left(t\right)\cdot \xi \left(t\right)$$

As $S\left(t\right)\to S_{\max}$, we have g(S(t))→0, and consequently:

(43) $$\lim_{S\left(t\right)\to S_{\max}}\frac{\partial \dot{S}}{\partial w_{\mathrm{drift}}}=0,\;\lim_{S\left(t\right)\to S_{\max}}\frac{\partial \dot{S}}{\partial w_{\mathrm{geo}}}=0$$

This indicates that as the setpoint approaches the saturation ceiling, the error rate of the PL becomes completely decoupled from the adaptive parameters. Regardless of how severe the directional drift $|d_{i}|$ or the geometric sensitivity ξ becomes, the system physically stops inflating the bound.

**Figure 12:**
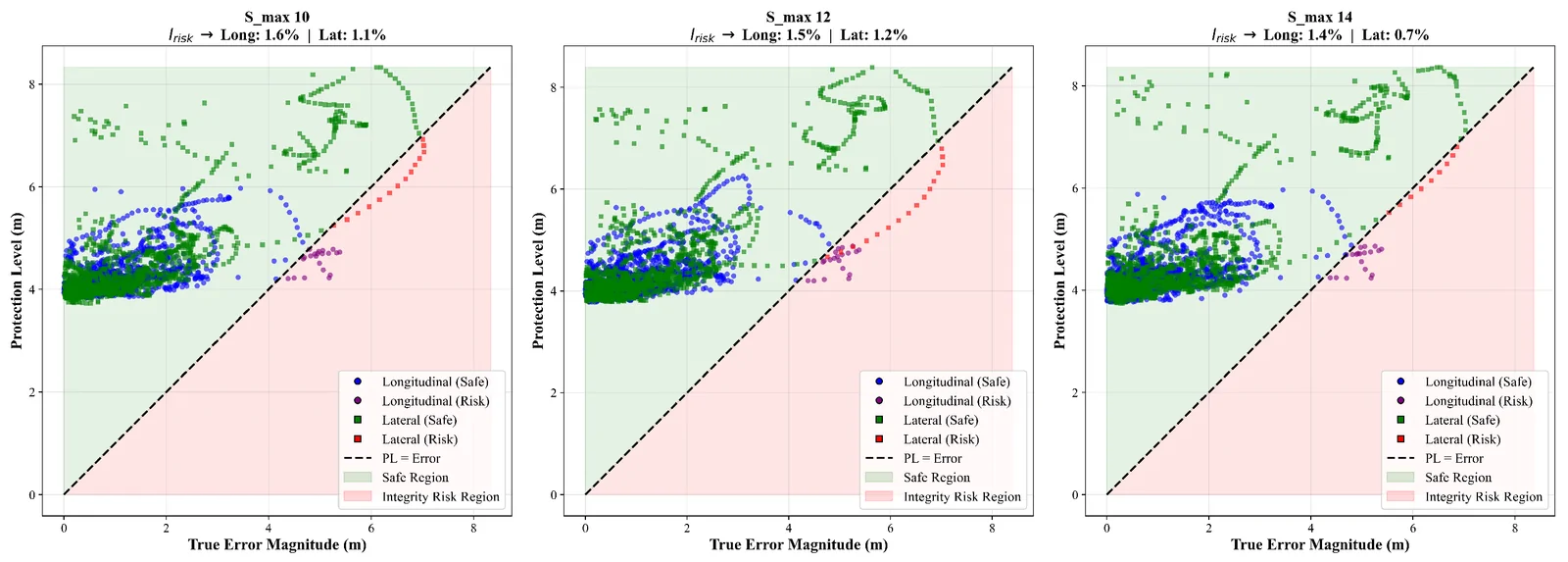
Stanford diagrams illustrating the impact of $S_{\max}$ on PL bounds and PL. From (**left**) to (**right**): $S_{\max}=10$, $S_{\max}=12$, and $S_{\max}=14$. Higher $S_{\max}$ delays saturation, allowing the PL to expand and capture extreme error peaks.

The Stanford diagrams in Figure 12 empirically validate this theoretical behavior by illustrating the direct impact of the saturation ceiling on system safety. As shown in the left panel, setting $S_{\max}=10$ causes the PL to prematurely cluster and flatline horizontally between 4 and 6 m on the y-axis. Because the saturation function g(S(t)) approaches zero at this ceiling, the system’s sensitivity to $w_{\mathrm{drift}}$ and $w_{\mathrm{geo}}$ is effectively nullified, preventing the PL from scaling with severe error spikes. Consequently, the true error pierces the safety boundary (crossing into the red region), yielding a 1.6% longitudinal and 1.1% lateral IR. By increasing $S_{\max}$ to 14 (right panel), the fraction S(t)/14 remains below unity for longer, maintaining the controller’s sensitivity to physical drift and geometric degradation. The Stanford diagram reflects this as the PL data points expand vertically, reaching up to approximately 8 m to successfully capture the heavy-tailed error excursions. This expansion reduces the longitudinal PL to 1.4% and significantly drops the lateral PL to 0.7%. While a larger $S_{\max}$ demonstrably improves adaptability during extreme degradation, it cannot be increased indefinitely without compromising nominal boundedness guarantees. This trade-off arises because the saturation ceiling $S_{\max}$ is currently fixed. A higher ceiling allows the PL to expand more during severe degradation, but makes it less tight in normal conditions. A lower ceiling keeps the bounds tighter, but may limit responsiveness when the environment is most challenging. Since the optimal ceiling depends on the operating conditions, future work will investigate online tuning of $S_{\max}$, allowing the system to adjust the ceiling as the environment changes.

The experimental results presented throughout this section substantiate all three claims. The proposed framework consistently produces smooth PLs, adapts effectively via entropy-driven gains, and enforces conservative asymmetric dynamics, achieving superior integrity performance across all tested urban scenarios.

## 6. Conclusions

This paper introduced a paradigm shift in PL calculation for multi-modal localization systems. Rather than relying on covariance estimates that assume Gaussian error distributions and require fault detection and exclusion steps, we reformulated integrity monitoring as a closed-loop control problem. The proposed framework computes an instantaneous error rate from inertial noise, kinematic slip, and LiDAR registration quality, then processes this rate through saturation-controlled setpoint dynamics and an adaptive PID controller with entropy-based gain scheduling. Asymmetric update laws enforce conservative behavior: PLs rise immediately when risk increases but decay slowly, requiring sustained evidence of safety before bounds relax.

Experimental validation on three UrbanNavDataset sequences demonstrated that traditional covariance-based methods exhibit integrity risks up to 46.2% in deep urban environments, effectively decoupling from physical reality. In contrast, the proposed framework achieved 0.0% integrity risk in medium-urban and low-urban conditions, and 2.3% under extreme deep-urban degradation. The minor breach observed in the deep-urban scenario reveals a fundamental limit of relative localization: when environments lack distinctive features and absolute positioning is unavailable for extended periods, no purely relative bounding algorithm can guarantee absolute safety. Nevertheless, the proposed framework degrades gracefully where traditional methods collapse entirely.

Future work will explore online tuning of $S_{\max}$ along with incorporating additional sensor modalities. The control-theoretic paradigm introduced here opens new directions for integrity monitoring beyond the limitations of statistical covariance-based methods.

## Data Availability

No new data were created or analyzed in this study. Data sharing is not applicable.

## Appendix A

**Algorithm A1** Protection level estimation via adaptive PID setpoint tracking
**Require:** ${\hat{\psi }}_{k}$: Current heading estimate $p_{k-1},p_{k}$: ES-EKF global position estimates $v_{k-1},a_{k},R_{k}$: IMU state and raw measurements $V_{k},J$: LiDAR scan points and ICP registration Jacobian $v_{\mathrm{gnss}}$: GNSS innovation vector (if available) **Ensure:** $PL_{\mathrm{long}},PL_{\mathrm{lat}}$ **Initialization (First Iteration Only):** 1: $PL_{i}\leftarrow K_{\mathrm{TIR}}\cdot \sigma_{\mathrm{base}}$, $S_{i}\leftarrow PL_{i},\;I_{i}\leftarrow 0,\;D_{i}\leftarrow 0$ for i∈{long,lat} **Main Loop:** 2: **for** each timestep *k* **do** **1. Directional Basis Projection** 3: $\hat{T}\leftarrow {\left[\cos\left({\hat{\psi }}_{k}\right),\sin\left({\hat{\psi }}_{k}\right)\right]}^{⊤},\;\hat{N}\leftarrow {\left[-\sin\left({\hat{\psi }}_{k}\right),\cos\left({\hat{\psi }}_{k}\right)\right]}^{⊤}$ **2. directional drift & Geometric Sensitivity** 4: $\Delta p\leftarrow p_{k}-p_{k-1}$, $\Delta p_{\mathrm{dr}}\leftarrow v_{k-1}\Delta t+\frac{1}{2}\left(R_{k}a_{k}-g\right)\Delta t^{2}$ 5: $d\leftarrow \Delta p-\Delta p_{\mathrm{dr}}$, $d_{\mathrm{long}}\leftarrow |d\cdot \hat{T}|,\;d_{\mathrm{lat}}\leftarrow \left|d\cdot \hat{N}\right|$ 6: $r_{\mathrm{geo}}\leftarrow \sqrt{\frac{1}{|V_{k}|}\sum_{v\in V_{k}}{∥p_{v}^{\mathrm{map}}-m\left(p_{v}^{\mathrm{scan}}\right)∥}_{2}}$, $\xi \leftarrow 1/\sqrt{\lambda_{\min}\left(J^{⊤}J\right)+\epsilon }$ **3. Adaptive Gain Scheduling** 7: Compute $H_{k}\in \left[0,1\right]$ from Shannon entropy of residual window $V_{k}$ 8: $K_{p}\leftarrow K_{p}^{0}\left(1+\alpha_{p}H_{k}\right)$, $K_{i}\leftarrow K_{i}^{0}\left(1-\alpha_{i}H_{k}\right)$, $K_{d}\leftarrow K_{d}^{0}\left(1-\alpha_{d}H_{k}\right)$ **4. Directional Updates** 9: **for** each i∈{long,lat} **do** 10: $\Gamma_{i}\leftarrow \sigma_{\mathrm{base}}+w_{\mathrm{drift}}\frac{|d_{i}|}{\Delta t}+w_{\mathrm{geo}}r_{\mathrm{geo}}\xi$ 11: $S_{i}\leftarrow S_{i}+\left[1-{\left(S_{i}/S_{\max}\right)}^{n}\right]\Gamma_{i}\Delta t$ 12: **if** $v_{\mathrm{gnss}}$ is available **then** 13: $v_{\mathrm{proj}}\leftarrow \left|v_{\mathrm{gnss}}\cdot \left(\hat{T}\;\mathrm{if}\;i=\mathrm{long}\;\mathrm{else}\;\hat{N}\right)\right|$ 14: $S_{i}\leftarrow \min\left(S_{i},\;v_{\mathrm{proj}}+K_{\mathrm{TIR}}\sigma_{\mathrm{gnss}}\right)$ 15: **end if** 16: $e_{i}\leftarrow S_{i}-PL_{i}$ 17: **if** $|I_{i}|<\gamma S_{\max}$ **or** $e_{i}\cdot I_{i}<0$ **then** 18: $I_{i}\leftarrow I_{i}+e_{i}\Delta t$ 19: **end if** 20: $\alpha_{\mathrm{filt}}\leftarrow \min\left(1.0,2\pi f_{c}\Delta t\right)$ 21: $D_{i}\leftarrow \alpha_{\mathrm{filt}}\frac{e_{i}-e_{i,\mathrm{prev}}}{\Delta t}+\left(1-\alpha_{\mathrm{filt}}\right)D_{i}$ 22: $u_{i}\leftarrow K_{p}e_{i}+K_{i}I_{i}+K_{d}D_{i}$ 23: $e_{i,\mathrm{prev}}\leftarrow e_{i}$ 24: *// Asymmetric Update and Clamping* 25: $PL_{i,k}=PL_{i,k-1}+\left\{\begin{array}{cc} u_{i,k}\Delta t, & \mathrm{if}\;u_{i,k}\geq 0\;(\mathrm{rising}:\;\mathrm{full}\;\mathrm{speed})\\ \alpha_{\mathrm{decay}}\cdot u_{i,k}\Delta t, & \mathrm{if}\;u_{i,k}<0\;(\mathrm{falling}:\;\mathrm{damped}) \end{array}\right.$ 26: $PL_{i}\leftarrow \max\left(K_{\mathrm{TIR}}\sigma_{\mathrm{base}}\Delta t,\;\min\left(PL_{i},S_{\max}\right)\right)$ 27: **end for** 28: **end for** 29: **return** $PL_{\mathrm{long}},PL_{\mathrm{lat}}$
